# Characterization of the nutritional status of university students, Medellín-Colombia, 2022

**DOI:** 10.15649/cuidarte.4317

**Published:** 2025-04-28

**Authors:** María Eugenia Muñoz-Galeano, Eliana Londoño-Cano, Carlos Andrés Vargas-Alzate

**Affiliations:** 1 Nutricionista Dietista, Magíster en Ciencias de la Alimentación y Nutrición Humana. Investigadora independiente. marunutricion@gmail.com Investigadora independiente Investigadora independiente Colombia marunutricion@gmail.com; 2 Nutricionista Dietista, Magíster en Ciencias de la Alimentación y Nutrición Humana. Grupo de investigación en Salud Familiar y Comunitaria, Facultad Ciencias de la Salud, Corporación Universitaria Remington Medellín, Colombia. eliana.londono@uniremington.edu.co Facultad Ciencias de la Salud, Corporación Universitaria Remington Corporación Universitaria Remington Medellín Colombia eliana.londono@uniremington.edu.co; 3 Gerente de Sistemas de Información en Salud. Magíster en Epidemiología. Grupo de Investigación en Neurociencias y Envejecimiento – GISAM, Facultad de Ciencias de la Salud, Corporación Universitaria Remington. Rehabilitación en Salud, Facultad de Medicina, Universidad de Antioquia. Medellín, Colombia. carlos.vargas01@uniremington.edu.co Facultad de Ciencias de la Salud, Corporación Universitaria Remington Corporación Universitaria Remington Medellín Colombia carlos.vargas01@uniremington.edu.co

**Keywords:** Nutritional Status, Non-communicable Diseases, Students, Sedentary lifestyle, Food Consumption, Estado Nutricional, Enfermedades no Transmisibles, Estudiantes, Sedentarismo, Consumo de Alimentos, Estado Nutricional, Doenças não Transmissíveis, Estudantes, Sedentarismo, Consumo de Alimentos

## Abstract

**Introduction:**

The rising prevalence of overweight and non-communicable chronic diseases (NCDs) represents a serious public health problem for young people in countries across the Americas.

**Objective:**

To analyze the nutritional status of students at a higher education institution in Medellín, Colombia, 2022.

**Materials and Methods:**

A cross-sectional descriptive study was conducted with 352 students. Sociodemographic information, nutritional status, and lifestyle habits were collected through surveys. Validated instruments were used to measure nutritional indicators. Data analysis included frequency distributions, summary measures, and statistical tests applied based on the assumption of normality.

**Results:**

31.82% of students were overweight, 22.16% had a high body fat percentage, and 19.32% were at cardiovascular risk. A statistical association was observed between BMI and both academic programs and age. While the consumption of natural foods was common, there was also a high intake of ultra-processed foods. Additionally, 62.78% of students engaged in adequate physical activity, yet 95.17% displayed sedentary behavior, and 39.77% had low handgrip strength. A potential positive correlation was identified between BMI, waist circumference, body fat percentage, and handgrip strength.

**Discussion:**

The study revealed a concerning nutritional profile among university students, marked by overweight, increased adiposity, and sedentary behavior, aligning with findings from similar studies conducted in this population across various regions. Although the frequent consumption of natural foods and engagement in adequate physical activity are positive aspects, they contrast with the high intake of ultra-processed foods and prevalent sedentary lifestyles—patterns consistent with research in Latin America.

**Conclusion:**

The university environment often increases students' risk of metabolic disorders, high consumption of ultra-processed foods, excess adiposity, and low physical activity levels, contributing to a nutritional profile that calls for attention and implementing preventive educational strategies.

## Resumen

**Introducción:**

El aumento del exceso de peso y de las enfermedades crónicas no transmisibles (ECNT) representa un grave problema de salud pública para los jóvenes de países de las Américas.

**Objetivo:**

Analizar el estado nutricional de los estudiantes de una institución de educación superior, Medellín-Colombia 2022.

**Materiales y métodos:**

Estudio descriptivo transversal con 352 estudiantes. Se recolectó información sociodemográfica, sobre el estado nutricional y los hábitos de vida mediante encuestas. Los instrumentos aplicados están validados para la medición de indicadores nutricionales. Se calcularon frecuencias, medidas de resumen y pruebas estadísticas según el supuesto de normalidad.

**Resultados:**

El 31,82% de los estudiantes presentó exceso de peso, 22,16% alto porcentaje de grasa y 19,32% riesgo cardiovascular. Se encontró una asociación estadística entre el IMC con el programa académico y la edad. Aunque el consumo de alimentos naturales fue frecuente, se encontró un alto consumo de ultraprocesados. El 62,78% presentó actividad física adecuada, el 95,17% sedentarismo y el 39,77% bajos niveles de fuerza prensil. Además, se identificó una posible correlación positiva entre el IMC, la circunferencia de la cintura, el porcentaje de grasa y la fuerza prensil.

**Discusión:**

El estudio mostró un perfil nutricional preocupante en estudiantes universitarios, caracterizado por el exceso de peso, adiposidad y sedentarismo, consistente con estudios de diferentes regiones realizados en la misma población. Aunque el consumo de alimentos naturales y la actividad física adecuada son factores positivos, estos contrastan con el alto consumo de ultraprocesados y el sedentarismo, hallazgos que coinciden con estudios en Latinoamérica.

**Conclusiones:**

El entorno en el que usualmente se encuentran los estudiantes universitarios aumenta el riesgo de alteraciones metabólicas, ya que el consumo de productos ultraprocesados, el exceso de adiposidad y los bajos niveles de actividad física, condicionan un perfil nutricional que requiere de estrategias educativas preventivas.

## Resumo

**Introdução:**

O aumento do excesso de peso e das doenças crônicas não transmissíveis (DCNT) representa um grave problema de saúde pública para os jovens em países das Américas.

**Objetivo:**

Analisar o estado nutricional dos estudantes de uma instituição de ensino superior em Medellín, Colômbia, 2022.

**Materiais e métodos:**

Estudo descritivo transversal com 352 estudantes. Informações sociodemográficas, estado nutricional e hábitos de vida foram coletados por meio de pesquisas. Os instrumentos aplicados são validados para mensuração de indicadores nutricionais. Frequências, medidas de resumo e testes estatísticos foram calculados de acordo com a suposição de normalidade.

**Resultados:**

31,82% dos estudantes apresentavam sobrepeso, 22,16% apresentavam alto percentual de gordura e 19,32% apresentavam risco cardiovascular. Foi apresentada associação estatística entre o IMC com o programa acadêmico e a idade. O consumo de alimentos naturais era frequente, porém o consumo de alimentos ultraprocessados era alto. 62,78% apresentaram atividade física adequada, 95,17% sedentarismo e 39,77% baixos níveis de força de preensão. Existe uma possível correlação positiva entre o IMC e a circunferência da cintura, a porcentagem de gordura e a força de preensão.

**Discussão:**

O estudo mostrou um perfil nutricional preocupante em universitários, com excesso de peso, adiposidade e sedentarismo, consistente com estudos de diferentes latitudes realizados na mesma população. Embora o consumo de alimentos naturais e a atividade física adequada sejam fatores positivos, eles contrastam com o alto consumo de alimentos ultraprocessados e o sedentarismo, que coincidem com estudos na América Latina.

**Conclusões:**

O ambiente em que os estudantes universitários costumam se encontrar aumenta o risco de distúrbios metabólicos, uma vez que o consumo de produtos ultraprocessados, o excesso de adiposidade e os baixos níveis de atividade física condicionam um perfil nutricional que requer estratégias educativas preventivas.

## Introduction

Excess weight and chronic non-communicable diseases (NCDs) represent significant public health challenges in the Americas, particularly among young people^1^. In the case of university students, this transitional stage into adulthood is often marked by adopting new habits, many of which negatively impact health. Limited access to nutritious foods, heavy academic workloads, financial constraints, and the extensive use of electronic devices contribute to increased sedentary behavior and high consumption of ultra-processed foods. These factors, combined with an environment that often neglects physical well-being, heighten the risk of metabolic disorders and the development of NCDs later in life^2^-^5^.

According to the World Health Organization (WHO), NCDs cause approximately 17 million deaths annually worldwide, demonstrating their devastating impact and the urgent need for effective preventive strategies^6^. Despite the existence of public policies and government initiatives aimed at addressing this issue, NCDs remain the leading causes of morbidity and mortality, with a concerning outlook that may worsen for future generations^6^. 

Studies in Latin America have reported a concerning rise in the prevalence of overweight and obesity among university students, with rates ranging between 45.0% and 51.0% in countries such as Chile, Peru, and Ecuador. However, this situation may vary depending on the social and economic context of each region^3^-^5^. Additionally, the literature describes a "double burden of malnutrition" in this adolescent population, characterized by the coexistence of nutritional deficiencies and excess weight. This imbalance stems from diets lacking essential nutrients while being high in calories from sugars and fats^7^,^8^. 

In Colombia, the situation is equally concerning. According to the National Survey of Nutritional Situation (ENSIN 2015), 57.0% of Colombian adults are overweight or obese, reflecting a sustained increase compared to previous studies^9^. In Medellín, the capital of the department of Antioquia and one of the main cities in the country, the prevalence of excess weight is 51.5%^10^. This alarming trend also affects younger populations and is driven by various social and economic factors, including unequal access to nutritious foods, rapid urbanization, and barriers to physical activity^11^,^12^. 

In the university context, students' sociodemographic and cultural characteristics in the city of Medellín play a crucial role in their nutritional profile. The region, known for its cultural and socioeconomic diversity, faces significant challenges regarding access to healthy food and promoting physical activity. The rising cost of living in recent years has led many young people to opt for more affordable food choices, often ultra-processed products that are calorie-dense but deficient in essential micronutrients^11^,^12^. Additionally, the demands of academic and work responsibilities contribute to a lack of time, fostering sedentary behaviors and limiting engagement in regular physical activities^13^. 

A sedentary lifestyle and a lack of moderate or vigorous physical activity have been identified as a significant risk factor in college students. According to the World Health Organization (WHO), this behavior can increase the risk of premature death by 20% to 30% compared to physically active individuals^14^,^15^. In Colombia, recent studies have shown that sedentary lifestyle levels among university students are alarmingly high, which contributes to a higher risk of cardiovascular and metabolic diseases^16^,^17^. 

The university environment, where students spend a substantial portion of their time, promotes healthy lifestyle habits and prevents metabolic disorders in young adults. Given the increasing burden of non-communicable diseases (NCDs), localized research is essential to assess students' nutritional status and lifestyle behaviors, enabling the development of targeted interventions. This study aimed to evaluate the nutritional status of students at a higher education institution in Medellín, Colombia. The findings provide evidence to design preventive strategies to promote healthier lifestyles and mitigate NCD risk within this vulnerable population. 

## Materials and Methods

A descriptive, cross-sectional study with an analytical scope was conducted among students from a higher education institution in Medellín, enrolled in the 2022 academic year. The sample size was calculated at 352 students, and the parameters were an expected prevalence of overweight people of 51%^18^, a confidence level of 95%, and a precision error of 5%. The sample was selected by convenience. Students from different academic programs, genders, and ages were included according to their availability to participate and who had current enrollment. Students who were pregnant, with pacemakers or prostheses, a history of hand surgery, and any condition that prevented adequate measurement (pain, recent surgery, among others) were excluded. 

Students were surveyed, and the data included sociodemographic variables (sex, age, marital status, socioeconomic status, ethnicity, and region of origin), nutritional status, and lifestyle habits. The data was obtained through individual sessions led by trained nutrition and dietetics students under the supervision of a professional nutritionist certified at Level II by the International Society for the Advancement of Kinanthropometry (ISAK). 

Validated instruments for population studies were used for each component, as appropriate: (i) Anthropometry: an adapted "ISAK full profile" form was used^19^. The variables taken into account were weight, height, waist circumference, skinfolds, body mass index (BMI), BMI/age, cardiovascular risk, and percentage of fat; (ii) Food consumption: the semi-quantitative food frequency questionnaire of Monsalve et al. was used^20^; (iii) Grip strength: The protocol of the American Society for Surgery of the Hand was applied. The classification was made according to the grip strength percentiles reported by Schlüssel et al. for adults and the Marrodán Serrano percentiles for adolescents^21^,^22^; (iv) Physical activity: The Global Physical Activity Questionnaire (GPAQ) was administered to determine participants' physical activity level and sedentary behaviors^23^. A description of the definition of variables, reference values, and equipment used for data collection is provided in Supplementary Table 1 (Table S1). 

All data was collected using structured forms in Google Forms and flat files in Microsoft Excel. 

Eligibility criteria were thoroughly applied to avoid bias, and the staff in charge of using the instruments received (i) theoretical-practical training in taking anthropometric measurements by a nutrition professional and (ii) training to apply the questionnaire on physical activity and food consumption. A pilot test was carried out with 20 students from the sample to validate the instruments, techniques, and data collection times. Technical sheets for the instruments were prepared, and the equipment calibration was validated according to the supplier's specifications. 

For statistical analysis, absolute and relative frequencies were calculated for qualitative variables, and medians with interquartile range (IQR) were obtained according to the Shapiro-Wilk test for quantitative variables. The association between nutritional status indicators and sociodemographic characteristics was determined using Pearson's chi-square, linear chi-square, Mann-Whitney U, and Kruskal-Wallis tests. Their association with nutritional status indicators of a quantitative nature was assessed using Spearman's correlation coefficient. P values less than 0.05 were considered significant, and the data were processed in SPSS, version 21. 

The study was approved by the bioethics committee of the higher education institution where it was conducted, study record number 07202, and was classified as minimal risk according to Resolution 8430 of 1994 of the Ministry of Health and Social Protection. Additionally, it adhered to the ethical standards outlined in the Declaration of Helsinki (1975). Informed consent was obtained from all participants after they were fully informed about the study objectives, the intended use of collected data, and the measures implemented to protect their privacy, including data anonymization. The data collected are available for free access and consultation on Zenodo^24^. 

## Results

A total of 352 students were included in the study. As shown in Table 1, the majority were women (76.14%) and fell within the 16-to-27-year age group (92.33%). Regarding socioeconomic status, 41.76% belonged to lower strata, while 53.13% were from middle strata. Geographically, 67.05% of the participants were from the Coffee Belt and Antioquia. 

**Table 1:** Sociodemographic characteristics of students of a higher education institution, Medellín-Colombia 2022

Sociodemographic characteristics	n
Sex	
Female	76.14 (268)
Male	23.86 (84)
Age ranges	
16 to 21	71.31 (251)
22 to 27	21.02 (74)
28 to 33	4.83 (17)
34 to 39	1.42 (5)
40 to 45	1.14 (4)
46 to 51	0.0 (0)
52 to 57	0.28 (1)
Age in completed years - Median (IQR)	20 (18-22)
Marital status	
Single	92.90 (327)
Married/common-law marriage	5.97 (21)
Widowed	0.28 (1)
Other	0.85 (3)
Socioeconomic status	
High	5.11 (18)
Middle	53.13 (187)
Low	41.76 (147)
Region	
Coffee Belt and Antioquia	67.05 (236)
Pacific	17.05 (60)
Caribbean	10.23 (36)
Central	3.41 (12)
Amazon and Orinoquia	2.27 (8)
Ethnicity	
No ethnic affiliation	70.74 (249)
Afro-descendant	25.00 (88)
Indigenous	3.13 (11)
Other	1.14 (4)
Academic Program	
Nutrition and Dietetics	44.03 (155)
Medicine	26.14 (92)
Veterinary Medicine	14.77 (52)
Nursing	6.60 (23)
Pharmacy Management	5.40 (19)
Others¥	3.13 (11)
Academic semester	
1	37.50 (132)
2	13.92 (49)
3	16.48 (58)
4	13.92 (49)
5	6.82 (24)
6	3.69 (13)
7	5.40 (19)
8	1.42 (5)
9	0.57 (2)
10	0.28 (1)

¥ Programs that did not exceed 1.00%: Business and financial management; Industrial engineering; Occupational health and safety engineering; Software development; Law; Accounting sciences; and Business and financial administration.

Among the students evaluated, 31.82% (n=112) were overweight, 22.16% (n=78) had a high percentage of body fat, and 19.32% (n=68) had elevated cardiovascular risk (Table 2). Women and students aged 19 to 30 were the most affected, exhibiting significantly higher frequencies of excess weight and body fat. A potential statistical association was identified between the academic program and body fat percentage (p = 0.002) and waist circumference classification (p = 0.040).

Quantitative measurement of anthropometric indicators showed possible statistical associations between BMI and age ranges, the percentage of fat, and variables such as sex, age range, marital status, and semester, and waist circumference with sex and age ranges (Table S2). 

**Table 2:** Comparison of anthropometric indicators and sociodemographic characteristics of students of a higher education institution, Medellín-Colombia 2022

Sociodemographic characteristics	BMI Classification				p-value	Fat percentage				p-value	Waist circumference classification		p-value
	Underweight n=23	Normal range n=217	Overweight n=91	Obesity n=21		Very low / Low / Thinness n=70	Adequate n=204	High Moderate / Excess n=70	High/ Obese n=8		No cardiovascular risk n=284	High cardiovascular risk n=68	
	% (n)	% (n)	% (n)	% (n)		% (n)	% (n)	% (n)	% (n)		% (n)	% (n)	
Sex					0.069					0.017			0.232
Female	91.30 (21)	76.50 (166)	75.82 (69)	57.14 (12)		74.29 (52)	80.39 (164)	70.00 (49)	37.50 (3)		77.46 (220)	70.59 (48)	
Male	8.70 (2)	23.50 (51)	24.18 (22)	42.86 (9)		25.71 (18)	19.61 (40)	30.00 (21)	62.50 (5)		22.54 (64)	29.41 (20)	
Age ranges					0.006**					0.239			0.180
16 to 21	86.96 (20)	75.12 (163)	61.54 (56)	57.14 (12)		68.57 (48)	74.51 (152)	65.71 (46)	62.50 (5)		72.54 (206)	66.18 (45)	
22 to 27	13.04 (3)	19.35 (42)	25.27 (23)	28.57 (6)		27.14 (19)	16.67 (34)	28.57 (20)	12.50 (1)		20.77 (59)	22.06 (15)	
28 to 33	0.00 (0)	3.69 (8)	7.69 (7)	9.52 (2)		4.29 (3)	5.39 (11)	2.86 (2)	12.50 (1)		4.58 (13)	5.88 (4)	
34 to 39	0.00 (0)	0.92 (2)	3.30 (3)	0.00 (0)		0.00 (0)	1.47 (3)	2.86 (2)	0.00 (0)		0.70 (2)	4.41 (3)	
40 to 45	0.00 (0)	0.46 (1)	2.20 (2)	4.76 (1)		0.00 (0)	1.47 (3)	0.00 (0)	12.50 (1)		1.06 (3)	1.47 (1)	
46 to 51	0.00 (0)	0.00 (0)	0.00 (0)	0.00 (0)		0.00 (0)	0.00 (0)	0.00 (0)	0.00 (0)		0.00 (0)	0.00 (0)	
52 to 57	0.00 (0)	0.46 (1)	0.00 (0)	0.00 (0)		0.00 (0)	0.49 (1)	0.00 (0)	0.00 (0)		0.35 (1)	0.00 (0)	
Marital status					0.225					0.652			0.601
Single	100 (23)	94.47 (205)	87.91 (80)	90.48 (19)		95.71 (67)	93.14 (190)	88.57 (62)	100 (8)		93.66 (266)	89.71 (61)	
Married/common-law marriage	0.00 (0)	5.07 (11)	9.89 (9)	4.76 (1)		4.29 (3)	5.88 (12)	8.57 (6)	0.00 (0)		5.28 (15)	8.82 (6)	
Widowed	0.00 (0)	0.46 (1)	1.10 (1)	4.76 (1)		0.00 (0)	0.49 (1)	2.86 (2)	0.00 (0)		0.70 (2)	1.47 (1)	
Other	0.00 (0)	0.00 (0)	1.10 (1)	0.00 (0)		0.00 (0)	0.49 (1)	0.00 (0)	0.00 (0)		0.35 (1)	0.00 (0)	
Socioeconomic status					0.119					0.967			0.547
High	4.35 (1)	5.53 (12)	4.40 (4)	4.76 (1)		7.14 (5)	4.41 (9)	4.29 (3)	12.50 (1)		4.93 (14)	5.88 (4)	
Middle	56.52 (13)	49.77 (108)	60.44 (55)	52.38 (11)		50.00 (35)	54.90 (112)	52.86 (37)	37.50 (3)		53.87 (153)	50.00 (34)	
Low	39.13 (9)	44.70 (97)	35.16 (32)	42.86 (9)		42.86 (30)	40.69 (83)	42.86 (30)	50.00 (4)		41.20 (117)	44.12 (30)	
Region					0.899					0.425			0.288
Coffee Belt and Antioquia	60.87 (14)	69.59 (151)	62.64 (57)	66.67 (14)		71.43 (50)	67.65 (138)	61.43 (43)	62.50 (5)		69.37 (197)	57.35 (39)	
Pacific	26.09 (6)	14.75 (32)	19.78 (18)	19.05 (4)		14.29 (10)	18.14 (37)	15.71 (11)	25.00 (2)		15.85 (45)	22.06 (15)	
Caribbean	13.04 (3)	10.14 (22)	10.99 (10)	4.76 (1)		10.00 (7)	9.80 (20)	12.86 (9)	0.00 (0)		10.21 (29)	10.29 (7)	
Central	0.00 (0)	3.69 (8)	3.30 (3)	4.76 (1)		2.86 (2)	3.43 (7)	4.29 (3)	0.00 (0)		2.82 (8)	5.88 (4)	
Amazon and Orinoquia	0.00 (0)	1.84 (4)	3.30 (3)	4.76 (1)		1.43 (1)	0.98 (2)	5.71 (4)	12.50 (1)		1.76 (5)	4.41 (3)	
Ethnicity					0.775					0.973			0.575
No ethnic affiliation	65.22 (15)	70.51 (153)	73.63 (67)	66.67 (14)		68.57 (48)	70.10 (143)	75.71 (53)	62.50 (5)		69.72 (198)	75.00 (51)	
Afro-descendant	30.43 (7)	25.81 (56)	19.78 (18)	33.33 (7)		25.71 (18)	25.49 (52)	21.43 (15)	37.50 (3)		25.35 (72)	23.53 (16)	
Indigenous	4.35 (1)	2.30 (5)	5.49 (5)	0.00 (0)		4.29 (3)	3.43 (7)	1.43 (1)	0.00 (0)		3.52 (10)	1.47 (1)	
Other	0.00 (0)	1.38 (3)	1.10 (1)	0.00 (0)		1.43 (1)	0.98 (2)	1.43 (1)	0.00 (0)		1.41 (4)	0.00 (0)	
Academic Program					0.122					0.002*			0.040*
Nutrition and Dietetics	47.83 (11)	49.31 (107)	34.07 (31)	28.57 (6)		50.00 (35)	43.63 (89)	41.43 (29)	25.00 (2)		46.83 (133)	32.35 (22)	
Medicine	34.78 (8)	22.58 (49)	28.57 (26)	42.86 (9)		25.71 (18)	25.49 (52)	28.57 (20)	25.00 (2)		25.00 (71)	30.88 (21)	
Veterinary Medicine	4.35 (1)	15.67 (34)	15.38 (14)	14.29 (3)		12.86 (9)	14.71 (30)	15.71 (11)	25.00 (2)		14.79 (42)	14.71 (10)	
Nursing	4.35 (1)	5.07 (11)	10.99 (10)	4.76 (1)		5.71 (4)	6.37 (13)	7.14 (5)	12.50 (1)		5.63 (16)	10.29 (7)	
Pharmacy Management	8.70 (2)	5.07 (11)	5.49 (5)	4.76 (1)		5.71 (4)	6.37 (13)	2.86 (2)	0.00 (0)		5.28 (15)	5.88 (4)	
Others¥	0.00 (0)	2.30 (5)	5.50 (5)	4.76 (1)		0.00 (0)	3.43 (7)	4.29 (3)	12.50 (1)		2.46 (7)	5.88 (4)	
Academic semester					0.205					0.139			0.113
1	52.17 (12)	40.09 (87)	31.87 (29)	19.05 (4)		40.00 (28)	41.18 (84)	24.29 (17)	37.50 (3)		39.08 (111)	30.88 (21)	
2	4.35 (1)	14.75 (32)	15.38 (14)	9.52 (2)		10.00 (7)	15.69 (32)	12.86 (9)	12.50 (1)		14.08 (40)	13.24 (9)	
3	17.39 (4)	14.75 (32)	19.78 (18)	19.05 (4)		14.29 (10)	16.18 (33)	21.43 (15)	0.00 (0)		16.20 (46)	17.65 (12)	
4	21.74 (5)	11.06 (24)	15.38 (14)	28.57 (6)		15.71 (11)	10.29 (21)	22.86 (16)	12.50 (1)		13.03 (37)	17.65 (12)	
5	4.35 (1)	7.83 (17)	5.49 (5)	4.76 (1)		8.57 (6)	7.35 (15)	4.29 (3)	0.00 (0)		7.75 (22)	2.94 (2)	
6	0.00 (0)	3.69 (8)	2.20 (2)	14.29 (3)		0.00 (0)	4.41 (9)	2.86 (2)	25.00 (2)		3.17 (9)	5.88 (4)	
7	0.00 (0)	4.61 (10)	8.79 (8)	4.76 (1)		8.57 (6)	2.45 (5)	10.00 (7)	12.50 (1)		4.23 (12)	10.29 (7)	
8	0.00 (0)	1.84 (4)	1.10 (1)	0.00 (0)		1.43 (1)	1.47 (3)	1.43 (1)	0.00 (0)		1.41 (4)	1.47 (1)	
9	0.00 (0)	0.92 (2)	0.00 (0)	0.00 (0)		1.43 (1)	0.49 (1)	0.00 (0)	0.00 (0)		0.70 (2)	0.00 (0)	
10	0.00 (0)	0.46 (1)	0.00 (0)	0.00 (0)		0.00(0)	0.49 (1)	0.00 (0)	0.00 (0)		0.35 (1)	0.00 (0)	

* Pearson Chi-square test. ** Linear Chi-square test. ¥ Business and financial management; Industrial engineering; Occupational health and safety engineering; Software development; Law; Accounting sciences; and Business and financial administration.

**Figure 1:**
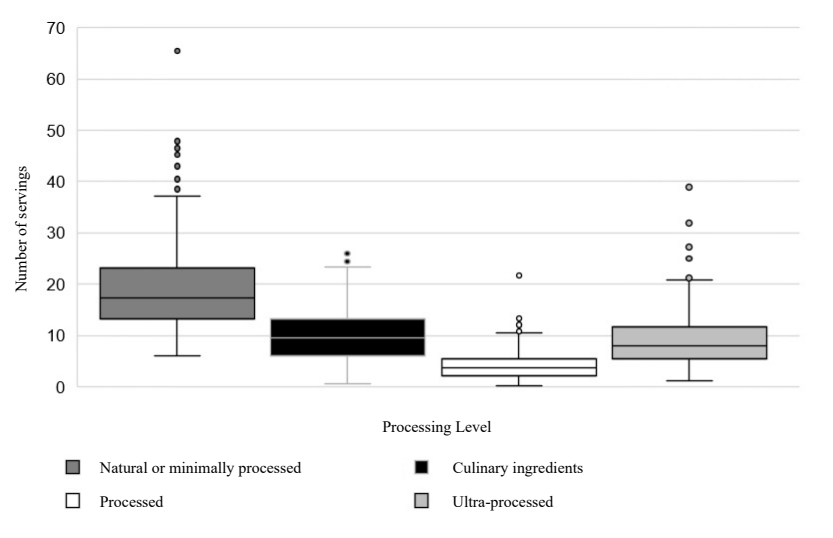
Distribution of portions consumed according to the level of food processing in students of a higher education institution, Medellín-Colombia 2022

Natural foods represented 44.92% (n = 6727) of the total intake, followed by culinary ingredients at 23.27% (n = 3485) and ultra-processed foods at 21.72% (n = 3253) (Table 3). This trend indicated a potential association with sex, as women consumed more natural foods, whereas men exhibited a greater preference for ultra-processed foods (p = 0.015). Figure 1 illustrates the distribution of portion consumption according to the degree of food processing, highlighting the more significant variability within the natural foods category. Additionally, a detailed analysis of portion consumption by food type revealed a high intake of cereals and flours, which was associated with sociodemographic characteristics (Tables S3 and S4). 

**Table 3:** Comparison of the number of portions consumed and sociodemographic characteristics of students at a higher education institution, Medellín-Colombia 2022

Sociodemographic characteristics	Natural or minimally processed n=6727	p-value	Culinary ingredients n=3485	p-value	Processed n=1511	p-value	Ultra-processed n=3253	p-value
	% (n)		% (n)		% (n)		% (n)	
Sex		0.143		0.756		0.666		0.015*
Female	73.63 (4954)		75.98 (2648)		75.99 (1149)		72.95 (2373)	
Male	26.37 (1774)		24.02 (837)		24.01 (363)		27.05 (880)	
Age ranges		0.694		0.165		0.261		0.457
16 to 21	70.44 (4738)		70.40 (2453)		69.72 (1054)		70.70 (2300)	
22 to 27	22.25 (1497)		22.16 (772)		21.41 (324)		22.04 (717)	
28 to 33	4.44 (299)		4.50 (157)		6.60 (100)		4.75 (154)	
34 to 39	1.42 (95)		1.64 (57)		1.00 (15)		1.48 (48)	
40 to 45	1.27 (85)		0.73 (26)		1.16 (18)		0.96 (31)	
46 to 51	0.00 (0)		0.00 (0)		0.00 (0)		0.00 (0)	
52 to 57	0.19 (13)		0.56 (20)		0.11 (2)		0.07 (2)	
Marital status		0.249		0.297		0.217		0.230
Single	93.33 (6279)		93.89 (3272)		93.06 (1407)		93.73 (3049)	
Married/common-law marriage	5.84 (393)		5.34 (186)		5.56 (84)		5.66 (184)	
Widowed	0.54 (36)		0.72 (25)		1.32 (20)		0.58 (19)	
Other	0.30 (20)		0.06 (2)		0.07 (1)		0.06 (2)	
Socioeconomic status		0.560		0.521		0.554		0.358
High	5.16 (347)		4.45 (155)		4.17 (63)		4.46 (145)	
Middle	51.68 (3477)		54.15 (1887)		53.31 (806)		54.44 (1771)	
Low	43.15 (2903)		41.38 (1442)		42.53 (643)		41.10 (1337)	
Region		0.617		0.349		0.283		0.721
Coffee Belt and Antioquia	66.93 (4503)		66.86 (2330)		66.40 (1004)		67.08 (2182)	
Pacific	16.93 (1139)		18.77 (654)		18.78 (284)		17.12 (557)	
Caribbean	10.66 (717)		9.81 (342)		9.92 (150)		11.10 (361)	
Central	2.93 (197)		2.81 (98)		3.31 (50)		2.86 (93)	
Amazon and Orinoquia	2.54 (171)		1.72 (60)		1.52 (23)		1.84 (60)	
Ethnicity		0.655		0.631		0.991		0.582
No ethnic affiliation	70.90 (4770)		70.70 (2464)		71.56 (1082)		71.47 (2325)	
Afro-descendant	25.34 (1705)		24.96 (870)		24.27 (367)		24.32 (791)	
Indigenous	2.84 (191)		3.50 (122)		3.04 (46)		2.77 (90)	
Other	0.91 (61)		0.83 (29)		1.12 (17)		1.44 (47)	
Academic Program		0.133		0.334		0.474		0.579
Nutrition and Dietetics	42.07 (2830)		43.96 (1532)		42.42 (641)		40.95 (1332)	
Medicine	26.31 (1770)		24.96 (870)		25.81 (390)		25.95 (844)	
Veterinary Medicine	16.25 (1093)		16.01 (558)		14.89 (225)		17.21 (560)	
Nursing	6.59 (443)		7.72 (269)		7.68 (116)		6.86 (223)	
Pharmacy Management	5.50 (370)		5.19 (181)		6.42 (97)		5.50 (179)	
Others¥	3.28 (221)		2.16 (75)		2.78 (42)		3.60 (117)	
Academic semester		0.443		0.609		0.400		0.595
1	36.44 (2452)		38.82 (1353)		40.94 (619)		39.90 (1298)	
2	14.36 (966)		13.77 (480)		13.23 (200)		14.42 (469)	
3	17.57 (1182)		16.56 (577)		15.28 (231)		16.17 (526)	
4	12.11 (815)		14.06 (490)		11.44 (173)		11.44 (372)	
5	6.96 (468)		5.37 (187)		7.54 (114)		5.75 (187)	
6	4.09 (275)		4.28 (149)		4.03 (61)		3.87 (126)	
7	5.65 (380)		5.19 (181)		4.50 (68)		5.41 (176)	
8	1.95 (131)		1.23 (43)		2.58 (39)		2.21 (72)	
9	0.54 (36)		0.52 (18)		0.33 (5)		0.58 (19)	
10	0.33 (22)		0.17 (6)		0.20 (3)		0.28 (9)	

* Mann-Whitney U test. ¥ Business and financial management; Industrial engineering; Occupational health and safety engineering; Software development; Law; Accounting sciences; and Business and financial administration

According to Table 4, 62.78% (n=221) of the students reported moderate or vigorous physical activity levels. However, 95.17% (n=335) presented sedentary behaviors, and 39.77% (n=140) had low levels of grip strength. Additionally, possible statistical associations were identified: the level of physical activity was related to sex, region of origin, academic program, and semester; sedentary behaviors, with marital status and academic program; and grip strength, with ethnicity.

Quantitative analysis of nutritional indicators identified the following statistical correlations: (i) positive between BMI, waist circumference, percentage of fat, and grip strength; and (ii) negative between the percentage of fat with grip strength (Rho= -0.126) and with physical activity level (Rho= -0.229). The level of physical activity showed possible statistical associations with BMI (p=0.044), categorized percentage of fat (p=0.023), cardiovascular risk (p=0.016), and sedentary lifestyle (p=0.001). Finally, the level of grip strength showed a possible association with the categorized percentage of fat (p=0.023).

**Table 4:** Comparison of the level of physical activity, sedentary lifestyle, and grip strength with the sociodemographic characteristics of students at a higher education institution, Medellín-Colombia 2022

Sociodemographic characteristics	Physical Activity Level (METS†)			p-value	Sedentarism		p-value	Dominant hand grip strength			p-value
	Light n=131	Moderate n=138	Vigotous n=83		No n=17	Yes n=335		Low n=140	Adequate n=145	Very good n=67	
	%(n)	%(n)	%(n)		%(n)	%(n)		%(n)	%(n)	%(n)	
Sex				<0.001*			0.257				0.112
Female	83.97 (110)	81.16 (112)	55.42 (46)		64.71 (11)	76.72 (257)		76.43 (107)	74.48 (108)	79.10 (53)	
Male	16.03 (21)	18.84 (26)	44.58 (37)		35.29 (6)	23.28 (78)		23.57 (33)	25.52 (37)	20.90 (14)	
Age ranges				0.202			0.123				0.112
16 to 21	76.34 (100)	73.91 (102)	59.04 (49)		52.94 (9)	72.24 (242)		73.57 (103)	67.59 (98)	74.63 (50)	
22 to 27	16.03 (21)	20.29 (28)	30.12 (25)		35.29 (6)	20.30 (68)		19.29 (27)	22.76 (33)	20.90 (14)	
28 to 33	4.58 (6)	2.17 (3)	9.64 (8)		5.88 (1)	4.78 (16)		4.29 (6)	6.21 (9)	2.99 (2)	
34 to 39	0.76 (1)	2.90 (4)	0.0 (0)		0.0 (0)	1.49 (5)		1.43 (2)	1.38 (2)	1.49 (1)	
40 to 45	1.53 (2)	0.72 (1)	1.20 (1)		5.88 (1)	0.90 (3)		1.43 (2)	1.38 (2)	0.00 (0)	
46 to 51	0.00 (0)	0.00 (0)	0.00 (0)		0.00 (0)	0.00 (0)		0.00 (0)	0.00 (0)	0.00 (0)	
52 to 57	0.76 (1)	0.00 (0)	0.00 (0)		0.00 (0)	0.30 (1)		0.00 (0)	0.69 (1)	0.00 (0)	
Marital status				0.395			0.019*				0.177
Single	92.37 (121)	94.93 (131)	90.36 (75)		76.47 (13)	93.73 (314)		94.29 (132)	91.03 (132)	94.03 (63)	
Married/common-law marriage	6.11 (8)	3.62 (5)	9.64 (8)		23.53 (4)	5.07 (17)		5.00 (7)	8.28 (12)	2.99 (2)	
Widowed	1.53 (2)	0.72 (1)	0.00 (0)		0.00 (0)	0.90 (3)		0.00 (0)	0.69 (1)	2.99 (2)	
Other	0.00 (0)	0.72 (1)	0.00 (0)		0.00 (0)	0.30 (1)		0.71 (1)	0.00 (0)	0.00 (0)	
Socioeconomic status				0.722			0.726				0.852
High	2.29 (3)	6.52 (9)	7.23 (6)		5.88 (1)	5.07 (17)		7.86 (11)	4.83 (7)	0.00 (0)	
Middle	54.20 (71)	44.93 (62)	65.06 (54)		58.82 (10)	52.84 (177)		52.14 (73)	55.17 (80)	50.75 (34)	
Low	43.51 (57)	48.55 (67)	27.71 (23)		35.29 (6)	42.09 (141)		40.00 (56)	40.00 (58)	49.25 (33)	
Region				0.008*			0.736				0.192
Coffee Belt and Antioquia	66.41 (87)	59.42 (82)	80.72 (67)		70.59 (12)	66.87 (224)		65.71 (92)	71.03 (103)	61.19 (41)	
Pacific	20.61 (27)	22.46 (31)	2.41 (2)		11.76 (2)	17.31 (58)		17.14 (24)	12.41 (18)	26.87 (18)	
Caribbean	9.16 (12)	11.59 (16)	9.64 (8)		11.76 (2)	10.15 (34)		8.57 (12)	12.41 (18)	8.96 (6)	
Central	3.05 (4)	2.90 (4)	4.82 (4)		0.00 (0)	3.58 (12)		5.00 (7)	2.76 (4)	1.49 (1)	
Amazon and Orinoquia	0.76 (1)	3.62 (5)	2.41 (2)		5.88 (1)	2.09 (7)		3.57 (5)	1.38 (2)	1.49 (1)	
Ethnicity				0.191			0.426				0.003*
No ethnic affiliation	64.12 (84)	70.29 (97)	81.93 (68)		88.24 (15)	69.85 (234)		79.29 (111)	68.28 (99)	58.21 (39)	
Afro-descendant	29.77 (39)	26.09 (36)	15.66 (13)		11.76 (2)	25.67 (86)		18.57 (26)	24.14 (35)	40.30 (27)	
Indigenous	4.58 (6)	2.90 (4)	1.20 (1)		0.00 (0)	3.28 (11)		2.14 (3)	5.52 (8)	0.00 (0)	
Other	1.53 (2)	0.72 (1)	1.20 (1)		0.00 (0)	1.19 (4)		0.00 (0)	2.07 (3)	1.49 (1)	
Academic Program				0.007*			0.001*				0.635
Nutrition and Dietetics	31.30 (41)	52.17 (72)	50.60 (42)		76.47 (13)	42.39 (142)		40.00 (56)	42.76 (62)	55.22 (37)	
Medicine	33.59 (44)	26.09 (36)	14.46 (12)		5.88 (1)	27.16 (91)		27.86 (39)	26.21 (38)	22.39 (15)	
Veterinary Medicine	18.32 (24)	9.42 (13)	18.07 (15)		0.00 (0)	15.52 (52)		16.43 (23)	14.48 (21)	11.94 (8)	
Nursing	9.92 (13)	3.62 (5)	6.02 (5)		0.00 (0)	6.87 (23)		5.71 (8)	7.59 (11)	5.97 (4)	
Pharmacy Management	3.82 (5)	6.52 (9)	6.02 (5)		11.76 (2)	5.07 (17)		7.14 (10)	5.52 (8)	1.49 (1)	
Others¥	3.05 (4)	2.16 (3)	4.81 (4)		5.88 (1)	3.00 (10)		2.84 (4)	3.45 (5)	2.98 (2)	
Academic semester				0.045**			0.867				0.403
1	41.98 (55)	32.61 (45)	38.55 (32)		47.06 (8)	37.01 (124)		36.43 (51)	39.31 (57)	35.82 (24)	
2	17.56 (23)	11.59 (16)	12.05 (10)		5.88 (1)	14.33 (48)		13.57 (19)	14.48 (21)	13.43 (9)	
3	14.50 (19)	18.84 (26)	15.66 (13)		5.88 (1)	17.01 (57)		13.57 (19)	17.24 (25)	20.90 (14)	
4	12.98 (17)	18.12 (25)	8.43 (7)		5.88 (1)	14.33 (48)		16.43 (23)	13.10 (19)	10.45 (7)	
5	4.58 (6)	8.70 (12)	7.23 (6)		17.65 (3)	6.27 (21)		7.14 (10)	6.90 (10)	5.97 (4)	
6	3.82 (5)	2.90 (4)	4.82 (4)		11.76 (2)	3.28 (11)		3.57 (5)	3.45 (5)	4.48 (3)	
7	3.82 (5)	5.80 (8)	7.23 (6)		5.88 (1)	5.37 (18)		7.14 (10)	3.45 (5)	5.97 (4)	
8	0.76 (1)	0.00 (0)	4.82 (4)		0.00 (0)	1.49 (5)		0.71 (1)	1.38 (2)	2.99 (2)	
9	0.00 (0)	0.72 (1)	1.20 (1)		0.00 (0)	0.60 (2)		0.71 (1)	0.69 (1)	0.00 (0)	
10	0.00 (0)	0.72 (1)	0.00 (0)		0.00 (0)	0.30 (1)		0.71 (1)	0.00 (0)	0.00 (0)	

* Pearson Chi-square test. ** Linear Chi-square test. ¥ Business and financial management; Industrial engineering; Occupational health and safety engineering; Software development; Law; Accounting sciences; and Business and financial administration. † Metabolic Equivalent of Task.

## Discussion

This study revealed a concerning nutritional profile among university students, which is marked by excess weight and a high cardiovascular risk. These factors indicate an imbalance in energy regulation, potentially increasing the risk of developing non-communicable diseases (NCDs) in the medium and long term. Additionally, the frequent consumption of foods rich in simple carbohydrates and deficient in fiber, combined with low levels of physical activity, contributes to the accumulation of body fat and compromised metabolic health. These findings underline an increased risk of NCDs in the lives of young university students. 

The nutritional profile of the students revealed a prevalence of excess weight, falling within an intermediate range compared to other studies conducted in Chile, Peru, Ecuador, Venezuela, the United States, and Mexico, which report values ranging from 16.7% to 55.4%^3^-^5^,^25^-^27^. This finding suggests that, while excess weight is a global problem, the prevalence observed in this population indicates that socioeconomic and cultural factors significantly influence the construction of the nutritional profile. Furthermore, the possible associations observed between BMI and variables such as age and academic program highlight the need for intervention strategies. These strategies should account for students' demographic and academic characteristics to enhance the prevention of non-communicable diseases (NCDs)^28^-^30^. 

A high percentage of students presented elevated body fat levels, which reflects a tendency towards the accumulation of adiposity, a risk factor in developing metabolic diseases. This finding aligns with previous studies in Latin America, confirming that abdominal obesity and excess body fat are common problems in university populations, regardless of the national context^31^-^33^. Additionally, consistent with previous research, a possible relationship was observed between the percentage of fat and sociodemographic variables, such as sex and age^34^. 

The identification of high cardiovascular risk in students underlines the importance of recognizing risk factors in this young population before chronic diseases develop. When comparing these results with other studies in Latin America, it is observed that in Argentina, Cuba, and Chile, the percentage of students with cardiovascular risk varies between 18.2% and 37.0%^29^,^35^,^36^. These figures highlight the importance of implementing early detection and intervention programs for modifiable risk factors to reduce the impact of cardiovascular diseases at an early age and in university environments, where lifestyle habits can influence future health. 

The dietary pattern observed among students indicates a high intake of cereals and sugary products, a concerning trend, given that these foods are rich in calories but low in essential nutrients. Although fruit and vegetable consumption met the recommendations of the Food Guidelines for the Colombian Population (GABAS in Spanish)^37^, these findings contrast with previous studies on university populations, where fruit and vegetable intake was lower. However, they align with prior research reporting a higher consumption of sweets^3^,^38^,^39^. Although the intake of most foods was in line with the recommendations, it is necessary to encourage greater consumption of legumes and reduce the intake of sweets since various studies have linked these habits with the development of insulin resistance and type 2 diabetes mellitus in the long term. Additionally, the high caloric intake from sweets and fats may exacerbate body adiposity and energy imbalance in this population, contributing to metabolic disorders^40^,^41^. 

Regarding the NOVA classification, the predominance of natural food consumption is a positive aspect for students' health, as these foods are associated with a lower risk of chronic diseases. This finding is consistent with departmental reports from Antioquia^42^. However, the high intake of ultra-processed foods raises concerns, given its association with an increased risk of excess weight, metabolic and psychological disorders, and impaired academic performance. This trend aligns with studies conducted in other Latin American countries^3^,^38^,^39^,^43^. The preference for fast foods and ultra-processed products in this population is often attributed to factors such as lack of time, socialization, and the beginning of independent life. These aspects should be considered in health interventions^38^. 

The widespread adherence to physical activity recommendations among students is a positive finding, as regular exercise is recognized as a protective factor against cardiometabolic diseases by enhancing insulin sensitivity, improving lipid profiles, and regulating blood pressure^44^. However, the high prevalence of sedentary behavior in this population remains a significant concern. Prolonged physical inactivity, often associated with electronic devices and academic demands, partially offsets the benefits of regular exercise^14^,^15^. Comparisons with other regions reveal that countries such as Mexico and Chile report low physical activity levels among university students, whereas Spain exhibits higher levels^27^,^45^,^46^. This contrast highlights the need for programs that promote regular exercise and reduce sedentary behaviors within the university environment. 

The low grip strength frequently observed in this population, particularly among women, underlines the importance of including this indicator as a tool for nutritional assessment, as it is an early marker of morbidity and mortality associated with cardiometabolic diseases^47^. These results are consistent with a study conducted in China, where men showed 65.0% more strength and about 13.0% more arm circumference than women^48^. This disparity highlights the need for interventions that promote physical activity and muscle strengthening in both genders, with a particular focus on women, who, due to biological and social factors, tend to have lower muscle mass levels compared to men. 

The limitations of the study include: i) The type of study and its exploratory nature of statistical associations do not allow the establishment of causal relationships; ii) The use of a non-probabilistic sampling technique limits the generalizability of the results to the entire study population and prevents extrapolation to other university populations. iii) Although some anthropometric indicators were measured, no biochemical assessments were conducted to provide a more comprehensive evaluation of nutritional status. The data on eating habits and physical activity levels were self-reported, which may introduce recall or social desirability bias. (v) The study did not explore the impact of psychosocial or emotional factors—such as academic stress, anxiety, and social pressure—on nutritional status, which could have contributed to a more comprehensive understanding of its determinants. (vi) Finally, as the analysis was conducted within a single educational institution in Medellín, the findings may not fully represent the diversity of the university environment across the city.

## Conclusions

This study comprehensively describes the nutritional profile of students from a higher education institution in Medellín. These students are characterized by high levels of excess weight, elevated cardiovascular risk, and significant adiposity, along with unhealthy dietary patterns and lifestyle behaviors. The excessive consumption of ultra-processed foods, widespread sedentary habits, and low grip strength are key factors that heighten the risk of developing metabolic disorders and non-communicable chronic diseases in the future. 

These findings also underscore the influence of the university environment, where time constraints, limited financial resources, and restricted access to healthy food options contribute to these issues. Additionally, the observed associations between sociodemographic variables and nutritional status highlight the need for personalized intervention strategies. 

Ultimately, implementing comprehensive health promotion initiatives targeting this population is crucial. Strategies should focus on nutritional education, encouraging regular physical activity, and reducing sedentary behavior. These interventions will enhance students' current well-being, help prevent metabolic complications, and reduce the long-term burden of chronic diseases. 

## Supplemental Material

**Table S1:** Description of the process for collecting the study variables

Variable	Description
Sociodemographic variables	Sociodemographic variables were collected in an Excel-type form. Date of birth was asked to estimate age, self-reported marital status (single, married, free union, divorced, widowed), self-recognition of the person as belonging to some ethnic group was considered, namely: Indigenous, Afro-descendant (black, mulatto, Afro-Colombian), others (ROM, Palenquero from San Basilio, Raizal from the archipelago), none of the above (those who did not consider themselves belonging to any ethnic group or race), according to the ENSIN 2010^49^.
	For the socioeconomic stratum, the classification defined by the National Administrative Department of Statistics (DANE in Spanish) at the national level (Colombia) was considered, which establishes within its methodology to classify the houses by the quality of the environment, the materials used in the construction and the public services offered. There are 6 categories: stratum 1 (low-low); stratum 2 (low); stratum 3 (medium-low); stratum 4 (medium), stratum 5 (medium-high); and stratum 6 (high)^50^.
	As for the region of origin, the department was taken into account and subsequently grouped by regions according to DANE: Coffee Belt and Antioquia, Pacific, Caribbean, Central, Amazon, and Orinoquia^51^. The degree and semester the students were studying at the university were also taken into account.
Anthropometric variables	Weight was measured using a Seca scale calibrated with a sensitivity of 100 g and a capacity of 200 kg; height was measured using a Seca stadiometer calibrated with a sensitivity of 0.1 cm and a capacity of 200 cm; waist circumference was measured using a Lufkin metal tape calibrated with a sensitivity of 0.1 cm and a capacity of 200 cm; fat folds were measured using a Slim Guide caliper with a precision of 0.5 mm with an opening of 80 mm and a pressure of 10 g/mm. The measurements were taken twice to verify that they were within the allowed variability: 100 g for weight, 0.5 cm for height, and 0.5 cm for waist circumference.
	Regarding nutritional status, anthropometric, dietary and functional indicators were taken into account, such as body mass index (BMI) for those over 18 years of age with cut-off points <18.5 kg/m2 for underweight, 18.5 to 24.9 kg/m2 for adequate weight, 25 to 29.9 kg/m2 for overweight and ≥30 kg/m2 for obesity; for adolescents (under 18 years of age) the BMI/Age indicator was taken into account, and the cut-off points were those of the World Health Organization (WHO), which establish overweight when the BMI is one standard deviation above the median of the growth reference, and obesity when this measurement is greater than two standard deviations above the median of the growth reference, which vary depending on the exact age of the individuals^52^,^53^. To assess cardiovascular risk based on waist circumference, the reference values of the World Diabetes Federation for adults were taken into account, where a measurement ≥80 cm or ≥90 cm indicates cardiovascular risk for women and men, respectively^54^, and for adolescents, the waist circumference percentiles in schoolchildren from Bogotá (Colombia) were used: FUPRECOL Study, which vary according to the exact age of the subjects evaluated^55^. Regarding the measurement of fat percentage, the sum of triceps, bicipital, subscapular, and suprailiac folds was taken into account, and for the classification of fat percentage, the proposal of Durnin and Womersley for adults was adopted, who define in women <20% thinness, 20-27% adequate, 27-34% excess, >34% obesity and in men <15% thinness, 15-22% adequate, 22-28% excess, >28% obesity; In the case of adolescents, the proposal of TG Lohman was taken into account, where for women <12% very low, 12-15% low, 15-25% optimal, 25-30% high moderate, 30-35.5% high, ≥35% very high, and for men <6% very low, 6-12% low, 12-20% optimal, 20-25% high moderate, 25-32% high, ≥32% very high^56^,^57^.
Food consumption	The semi-quantitative food frequency questionnaire by Monsalve et al. was used, and a photo album designed to estimate the portion of each food consumed by the students was added. For the nutritional content, the nutrient information in the foods (RINAS in Spanish) was taken into account, and for the intake of a healthy diet, the Food Guidelines for the Colombian Population (GABAS in Spanish) and the NOVA classification of foods^58^-^61^.
Variable grip strength	A properly calibrated Jamar® digital hand dynamometer SP-5030JD was used, applying the protocol of the American Society for Surgery of the Hand, and the classification was made according to the percentiles of grip strength reported by Schlüssel et al. for adults and the percentiles of Marrodán Serrano for adolescents^62^,^63^; the maximum grip strength of the dominant hand was taken into account for the analysis. The cut-off points were: Low grip strength: <25th percentile, Adequate grip strength: between the 25th and 95th percentile, Excellent grip strength: >95th percentile.
Physical activity variables	The Global Physical Activity Questionnaire (QPAQ)^64^ was applied to determine the level of physical activity and sedentary behavior as follows: Light or insufficient activity: Less than 600 MET-min/week. Moderate activity: 600 to 2999 MET-min/week. Vigorous activity: more than 3000 MET-min/week). Sedentary behaviors refer to the total daily time a person spends sitting or reclining (excluding time spent sleeping). A person was considered to have sedentary behaviors if they spent more than 7 hours sitting or reclining.

**Table S2:** Summary measures of nutritional status and physical activity according to sociodemographic characteristics of university students, Medellín 2022

Sociodemographic characteristics	Waist circumference (cm)		p-value	BMI (m/t2)		p-value	Fat percentage		p-value	Maximum value of dominant hand grip strength (Kg)		p-value	METS		p-value
	Median	IQR		Median	IQR		Median	IQR		Median	IQR		Median	IQR	
Sex			**<0.001***			0.167			**<0.001***			**<0.001***			**<0.001* **
Female	70.73	66.38 - 77.33		22.95	20.84 - 25.44		22.23	18.59 - 25.13		27.40	24.30 - 31.80		720	300 - 2160	
Male	78.45	74.20 - 86.85		23.11	21.14 - 26.27		18.41	14.73 - 22.53		45.10	40.10 - 51.75		2600	580 - 5980	
Age ranges			0.021**			**0.004****			0.063			0.082			0.093
16 to 21	71.70	66.95 - 78.35		22.68	20.59 - 25.16		21.11	17.46 - 24.11		29.30	25.00 - 34.80		720	360 - 2400	
22 to 27	74.20	70.00 - 83.20		23.36	21.79 - 26.31		22.30	17.25 - 25.79		32.65	27.30 - 41.30		1790	400 - 4200	
28 to 33	76.00	68.00 - 80.00		25.22	23.21 - 26.98		22.65	18.69 - 23.91		29.60	25.90 - 45.40		1200	360 - 4440	
34 to 39	81.65	77.00 - 82.35		26.31	24.63 - 27.93		26.46	23.62 - 28.71		30.60	26.60 - 39.00		720	720 - 840	
40 to 45	76.98	75.58 - 93.28		25.46	24.50 - 30.76		23.72	22.33 - 27.13		28.75	24.35 - 30.55		680	480 - 16960	
46 to 51	-	-		-	-		-	-		-	-		-	-	
52 to 57	78.05	78.05 - 78.05		23.34	23.34 - 23.34		21.90	21.90 - 21.90		26.60	26.60 - 26.60		0	0 - 0	
Marital status			0.279			0.073			0.020**			0.469			0.566
Married/common-law marriage	77.00	72.00 - 81.65		24.63	23.34 - 25.96		24.07	22.65 - 26.45		29.40	26.60 - 32.30		720	240 - 4320	
Other	78.00	69.30 - 97.00		27.13	19.65 - 30.33		26.96	20.03 - 30.51		29.60	29.00 - 31.50		400	400 - 1920	
Single	72.15	67.50 - 79.00		22.86	20.88 - 25.49		21.46	17.45 - 24.23		30.10	25.40 - 37.40		840	400 - 2800	
Widowed	74.25	74.25 - 74.25		24.99	24.99 - 24.99		21.31	21.30 - 21.30		20.70	20.70 - 20.70		800	800 - 800	
Socioeconomic status			0.272			0.517			0.539			0.642			0.189
High	72.50	68.05 - 77.15		23.18	22.04 - 25.62		22.65	17.18 - 24.94		27.80	24.40 - 36.90		1590	720 - 4200	
Middle	71.95	66.15 - 78.50		22.81	20.56 - 25.41		20.64	17.31 - 24.19		30.60	25.40 - 36.10		720	360 - 2160	
Low	73.50	68.60 - 79.25		23.32	21.18 - 26.01		21.76	17.92 - 24.59		29.40	25.50 - 37.30		900	300 - 3360	
Region			0.170			0.309			0.156			0.461			0.073
Amazon and Orinoquia	79.58	75.65 - 84.13		25.24	24.17 - 27.03		26.85	23.26 - 27.82		26.70	25.60 - 33.00		1280	680 - 3660	
Caribbean	70.70	67.73 - 80.58		22.64	20.48 - 25.44		21.08	18.26 - 23.55		29.90	26.20 - 42.15		992	448 - 2540	
Coffee Belt and Antioquia	76.25	69.90 - 81.30		23.20	22.69 - 25.73		21.43	17.64 - 25.42		28.70	23.20 - 42.85		1020	360 - 8120	
Central	72.83	67.93 - 78.33		22.97	21.07 - 25.43		21.51	17.45 - 24.37		30.10	25.45 - 36.30		1080	320 - 3360	
Pacific	70.98	66.18 - 79.45		23.40	20.37 - 26.29		22.16	17.62 - 24.98		29.40	24.40 - 34.50		600	330 - 1310	
Ethnicity			0.176			0.722			0.943			0.756			0.030**
Afro-descendant	70.53	66.78 - 77.53		22.49	20.84 - 25.17		21.53	18.02 - 24.29		30.90	25.35 - 34.50		664	240 - 1760	
Indigenous	72.50	66.25 - 81.10		23.77	20.56 - 25.96		20.26	17.11 - 24.75		30.10	25.90 - 41.30		500	400 - 1200	
Other	70.43	67.85 - 78.80		21.23	20.29 - 24.02		21.99	17.04 - 23.78		38.00	27.90 - 46.25		640	300 - 3620	
No ethnic affiliation	74.05	68.00 - 79.50		23.13	21.08 - 25.73		21.71	17.59 - 24.67		29.40	25.40 - 39.00		1080	400 - 3180	
Academic Program			0.052			0.128			0.851			0.478			0.010**
Nutrition and Dietetics	70.95	66.60 - 77.30		22.83	28.82 - 24.79		21.66	17.66 - 24.40		29.60	25.40 - 34.40		1440	500 - 3680	
Medicine	73.58	67.70 - 80.78		22.81	20.93 - 25.93		21.58	17.39 - 24.36		29.20	25.25 - 37.40		600	170 - 1440	
Veterinary Medicine	75.28	69.65 - 78.65		23.23	21.89 - 26.01		21.35	17.39 - 24.63		31.30	26.10 - 42.15		780	360 - 3120	
Nursing	76.90	68.00 - 81.50		24.63	22.27 - 26.43		23.27	18.37 - 26.42		28.50	26.20 - 34.50		560	320 - 1600	
Pharmacy Management	74.25	69.00 - 78.35		21.93	19.00 - 25.52		21.31	16.24 - 23.62		29.40	22.10 - 39.60		800	400 - 3360	
Others	77.65	68.00 - 87.80		25.34	21.21 - 27.98		20.37	17.46 - 23.45		30.70	26.00 - 53.90		1440	400 - 3740	
Academic semester			0.330			0.347			**0.013** **			0.934			0.342
1	71.10	66.55 - 78.05		22.76	20.18 - 24.84		20.04	17.04 - 23.20		29.30	24.95 - 36.30		740	340 - 2880	
2	73.30	68.80 - 78.50		23.23	21.42 - 25.92		21.83	18.24 - 24.59		31.00	25.50 - 39.20		720	360 - 2520	
3	74.00	67.55 - 79.65		23.48	21.04 - 26.41		21.73	18.31 - 24.91		30.90	25.90 - 37.20		1112	400 - 2560	
4	74.30	69.10 - 82.50		23.49	21.30 - 26.77		23.05	19.64 - 26.56		30.10	26.30 - 35.10		764	160 - 1320	
5	71.25	66.20 - 77.43		22.22	20.89 - 24.83		21.61	19.04 - 25.33		29.60	25.60 - 32.50		860	580 - 3000	
6	76.50	69.00 - 87.60		24.28	21.97 - 29.33		24.36	21.90 - 28.36		30.30	27.50 - 37.80		1740	240 - 6360	
7	78.40	68.10 - 87.75		23.98	21.81 - 27.61		21.76	17.18 - 26.79		31.90	23.10 - 41.50		1320	500 - 6000	
8	71.25	71.05 - 75.60		22.25	22.18 - 23.31		23.27	20.95 - 24.75		29.10	28.00 - 34.10		5360	3740 - 6240	
9	68.50	65.35 - 71.65		21.57	20.63 - 22.51		20.91	18.50 - 23.31		25.60	18.10 - 33.00		2540	1080 - 4000	
10	72.35	72.35 - 72.35		23.48	23.47 - 23.47		22.79	22.78 - 22.78		22.78 - 22.78	25.80 - 25.80		2200	2200 - 2200	

Significant p values in bold * Mann-Whitney U ** Kruskal Wallis

**Table S3:** Food consumption by sex of university students, Medellín 2022

Food groups	Sex									p-value
	Female			Male			Total			
	Median	25th percentile	75th percentile	Median	25th percentile	75th percentile	Median	25th percentile	75th percentile	
Dairy	2.80	1.30	4.60	3.00	1.60	4.60	2.90	1.40	4.60	0.457
Meats and eggs	3.40	2.34	4.77	3.90	2.25	5.62	3.49	2.31	5.07	0.088
Legumes	0.60	0.20	0.80	0.60	0.20	0.90	0.60	0.20	0.90	0.414
Cereals and flours	6.15	4.40	8.15	7.20	4.45	11.25	6.25	4.40	8.45	0.055
Fruits, vegetables and greens	6.07	4.13	9.62	6.38	4.18	11.19	6.18	4.13	9.97	0.363
Monounsaturated fats	0.57	0.27	1.50	0.57	0.18	2.16	0.57	0.24	1.71	0.919
Polyunsaturated fats	1.12	0.07	4.77	1.02	0.14	5.00	1.07	0.10	5.00	0.338
Unsaturated fats	3.45	1.16	6.00	4.26	1.32	6.50	3.55	1.18	6.04	0.138
Saturated and trans fats	1.00	0.39	3.00	1.22	0.29	0.29	1.00	0.30	3.00	0.925
Sweets and desserts	4.90	2.80	7.60	5.75	3.25	8.65	5.00	3.00	7.70	0.199
Liquors	0.20	0.00	0.40	0.10	0.00	0.50	0.20	0.00	0.40	0.953
Natural or minimally processed	17.00	13.25	21.85	17.45	13.20	28.55	17.15	13.20	23.25	0.143
Culinary ingredients	9.50	6.00	13.00	9.35	6.25	13.60	9.50	6.05	13.30	0.756
Processed	3.60	2.10	5.60	3.70	2.55	5.35	3.60	2.20	5.55	0.666
Ultra-processed	7.90	5.35	11.00	8.80	6.60	13.40	7.90	5.50	11.65	0.015*

Significant p values in bold * Mann-Whitney U

**Table S4:** Food consumption according to NOVA classification according to sociodemographic characteristics of university students, Medellín 2022

Sociodemographic characteristics	Natural or minimally processed			Culinary ingredients			Processed			Ultra-processed		
	Median	25th percentile	75th percentile	Median	25th percentile	75th percentile	Median	25th percentile	75th percentile	Median	25th percentile	75th percentile
Sex												
Female	17.00	13.25	21.85	9.50	6.00	13.00	3.60	2.10	5.60	7.90	5.35	11.00
Male	17.45	13.20	28.55	9.35	6.25	13.60	3.70	2.55	5.35	8.80	6.60	13.40
Age ranges												
16 to 21	17.10	13.40	23.00	9.00	6.10	12.40	3.60	2.10	5.40	7.90	5.50	11.90
22 to 27	17.75	12.90	23.70	10.65	6.00	14.00	3.75	2.20	5.50	8.45	5.20	11.30
28 to 33	15.50	13.60	21.30	8.50	5.40	12.60	5.20	4.10	6.50	8.10	6.40	10.70
34 to 39	23.20	12.30	25.60	12.00	9.10	13.60	3.40	2.40	3.40	10.90	6.30	13.00
40 to 45	20.25	18.95	23.65	4.35	2.25	10.50	4.00	1.35	7.40	5.00	3.05	12.60
46 to 51	-	-	-	-	-	-	-	-	-	-	-	-
52 to 57	12.90	12.90	12.90	19.50	19.50	19.50	1.70	1.70	1.70	2.40	2.40	2.40
Marital status												
Married/common-law marriage	17.90	13.80	25.50	8.30	4.60	12.30	3.40	2.20	6.00	7.50	5.00	12.30
Other	12.30	9.50	13.80	8.70	5.50	11.00	4.40	3.60	11.50	5.80	4.70	8.00
Single	17.20	13.20	23.30	9.70	6.20	13.40	3.70	2.20	5.40	8.10	5.60	11.70
Widowed	20.00	20.00	20.00	2.30	2.30	2.30	0.50	0.50	0.50	2.30	2.30	2.30
Socioeconomic status												
High	17.90	13.40	23.20	8.50	4.30	12.90	3.50	1.30	4.60	7.15	4.00	10.30
Middle	16.70	13.30	21.70	10.00	6.10	14.00	3.70	2.20	5.70	8.00	5.60	12.40
Low	17.60	13.20	24.10	9.00	6.00	12.60	3.50	2.10	5.40	8.00	5.60	11.40
Region												
Amazon and Orinoquia	19.50	15.20	26.85	7.95	6.90	8.55	2.65	1.60	3.50	6.75	5.50	8.90
Caribbean	16.70	13.55	26.45	9.45	9.45	12.00	3.60	1.90	5.30	8.50	5.50	13.05
Coffee Belt and Antioquia	15.35	11.25	20.65	8.50	4.40	12.10	4.25	1.50	6.35	7.20	5.35	8.70
Central	16.95	13.25	22.85	9.50	5.65	13.55	3.55	2.20	5.40	7.95	5.50	11.75
Pacific	17.85	12.80	24.10	11.00	6.75	15.35	4.40	2.95	6.15	8.05	5.75	11.00
Ethnicity												
Afro-descendant	18.15	12.90	24.60	9.10	6.00	13.00	3.50	2.10	5.30	7.90	5.40	11.30
Indigenous	16.60	13.60	19.80	11.40	4.70	16.00	3.80	2.20	6.90	8.20	4.10	9.10
Other	13.70	11.60	19.10	6.50	5.60	8.80	3.10	1.80	6.60	11.85	6.80	16.80
No ethnic affiliation	16.70	13.40	22.70	9.60	6.10	13.40	3.70	2.20	5.60	7.90	5.70	11.80
Academic Program												
Nutrition and Dietetics	16.80	12.90	21.50	9.20	5.50	13.30	3.50	1.90	5.30	7.60	5.20	10.70
Medicine	16.60	13.35	24.75	9.00	5.85	12.70	3.60	2.25	5.35	7.95	5.45	11.20
Veterinary Medicine	17.80	13.70	26.55	10.30	7.50	13.65	3.90	2.30	5.30	9.35	6.20	13.45
Nursing	19.10	14.00	26.50	12.00	7.10	17.00	4.80	3.00	6.50	6.80	5.30	12.70
Pharmacy Management	15.70	14.40	25.20	8.50	5.10	14.60	4.50	2.30	7.50	8.50	6.00	11.60
Business administration and finance	14.80	14.80	14.80	6.10	6.10	6.10	2.50	2.50	2.50	9.80	9.80	9.80
Accounting sciences	12.70	12.70	12.70	12.00	12.00	12.00	4.60	4.60	4.60	8.40	8.40	8.40
Law	11.50	11.50	11.50	4.10	4.10	4.10	2.10	2.10	2.10	7.80	7.80	7.80
Software development	7.60	7.60	7.60	5.20	5.20	5.20	0.80	0.80	0.80	7.50	7.50	7.50
Business and financial management	24.60	19.60	30.10	5.00	3.10	10.00	3.70	3.10	6.20	12.40	8.90	15.40
Occupational health and safety engineering	15.30	12.40	18.20	6.50	1.10	11.90	6.50	2.00	11.00	15.10	8.10	22.10
Industrial engineering	34.75	30.50	39.00	8.30	7.60	9.00	3.00	1.10	4.90	8.10	7.20	9.00
Academic semester												
1	16.35	13.40	22.10	10.00	5.60	14.05	4.20	2.25	6.15	8.20	5.50	12.35
2	17.20	12.60	23.00	9.30	7.10	13.00	3.60	2.10	5.10	7.60	6.20	12.30
3	19.05	13.80	24.80	9.50	6.00	12.60	3.40	2.70	5.10	7.85	5.20	11.20
4	15.40	11.50	20.20	9.70	6.40	13.00	3.40	1.70	5.10	7.10	5.10	9.50
5	18.80	15.45	23.65	6.90	4.65	10.00	3.55	2.10	6.50	7.30	5.20	9.75
6	17.90	12.30	22.00	12.10	8.50	14.00	4.20	3.50	5.20	8.70	7.50	12.30
7	17.60	13.90	24.00	9.90	5.30	13.70	3.10	1.70	5.10	8.60	5.70	11.90
8	16.60	11.50	26.40	6.20	6.10	13.40	5.20	3.40	6.20	7.80	5.90	17.00
9	18.15	17.60	18.70	9.05	8.60	9.50	2.25	0.80	3.70	9.25	8.00	10.50
10	21.80	21.80	21.80	6.30	6.30	6.30	2.70	2.70	2.70	9.10	9.10	9.10
